# Novel *GJC2* and *OBSCN* variants co-segregating in a Chinese primary lymphedema pedigree

**DOI:** 10.1186/s13023-026-04196-7

**Published:** 2026-01-13

**Authors:** Xiaoqian Shi, Gang Wang, Shang Ju, Rui Huang, Runlin Z. Ma, Fuguang Liu, Hui Li, Jiawei Jin

**Affiliations:** 1 Medical Research Center, Beijing Chao-Yang Hospital, Capital Medical University, No.5 Jingyuan Road, Beijing Chaoyang Hospital Jingxi Campus, Beijing, 100043 China; 2 Department of Peripheral Vascular, Dongzhimen Hospital, Beijing University of Chinese Medicine, Beijing, 100010 China; 3 State Key Laboratory of Molecular Developmental Biology, Institute of Genetics and Developmental Biology, Chinese Academy of Sciences, Beijing, 100101 China; 4 Shacheng Town Health Center, Huailai County, Hebei, 075400 China; 5 Department of Respiratory and Critical Care Medicine, The First Hospital of Hebei Medical University, Hebei, 050023 China

**Keywords:** Primary lymphedema, *GJC2*, *OBSCN*, Linkage disequilibrium

## Abstract

**Background:**

Primary lymphedema (PL) is a genetically heterogeneous disorder of the lymphatic system. Despite the identification of numerous monogenic causes, the genetic etiology of many familial cases remains elusive, often exhibiting marked phenotypic variability that suggests non-Mendelian or complex inheritance patterns.

**Results:**

We investigated a four-generation Chinese pedigree presenting with autosomal dominant generalized lymphedema affecting all four limbs, with marked phenotypic variability. Whole-exome sequencing in eight key family members, followed by Sanger sequencing in all 26 available relatives, identified two novel heterozygous missense variants that co-segregated with the disease: *GJC2* c.287G > T (p.Gly96Val) and *OBSCN* c.22393T > A (p.Phe7465Ile). The *GJC2* variant affects the second transmembrane domain of connexin 47, whereas the *OBSCN* variant lies in immunoglobulin-like domain 55 of obscurin, a large cytoskeletal signaling protein not previously implicated in lymphedema. In silico structural modeling predicted that both variants alter local packing and compromise protein stability. Haplotype analysis revealed that these two genes, located approximately 210 kb apart on chromosome 1q42.13, reside on a shared haplotype block with linkage disequilibrium, consistent with co-inheritance as a single pathogenic haplotype.

**Conclusions:**

To our knowledge, this is the first report linking *OBSCN* to human lymphedema. We propose a synergistic oligogenic model in which *GJC2* disruption impairs lymphatic pumping coordination, while the co-segregating *OBSCN* variant compromises cytoskeletal integrity and RhoA signaling in lymphatic endothelial cells; together these effects could explain the early-onset, severe phenotype observed in this family. These findings expand the genetic spectrum of PL and highlight the importance of considering closely linked modifier genes in complex inheritance patterns.

**Supplementary Information:**

The online version contains supplementary material available at 10.1186/s13023-026-04196-7.

## Background

Primary lymphedema (PL) is a heterogeneous group of genetic and developmental disorders characterized by developmental aplasia or functional failure of the lymphatic vascular system. The lymphatic system plays an indispensable role in maintaining tissue fluid homeostasis, immune surveillance, and dietary lipid absorption. When this system is compromised, protein-rich interstitial fluid accumulates in the subcutaneous tissues, leading to chronic swelling (lymphedema), progressive adipose tissue deposition, and secondary fibrosis. Clinically, PL poses a significant burden, often resulting in limb deformities, recurrent erysipelas or cellulitis, and a profound impact on quality of life.

The genetic basis of PL is complex and follows Mendelian inheritance patterns in familial cases, predominantly autosomal dominant, although autosomal recessive and X-linked forms exist [1]. The classification of PL has traditionally relied on the age of onset: congenital lymphedema (Milroy disease, onset at birth), lymphedema praecox (Meige disease, onset at puberty), and lymphedema tarda (onset after age 35) [2]. However, the advent of next-generation sequencing (NGS) has shifted the nosology towards a molecular-based classification. To date, over 20 causal genes have been identified, most of which govern specific stages of lymphangiogenesis. For instance, *FLT4* (VEGFR3) and *VEGFC* regulate the initial sprouting of lymphatic vessels, while *FOXC2* and *GATA2* are critical for the maturation of lymphatic valves. Despite these advances, a significant proportion of familial PL cases remain genetically unsolved, or exhibit variable expressivity that single-gene models fail to explain, suggesting the involvement of modifier genes or oligogenic inheritance [3].

Among the established PL-associated genes, gap junction protein gamma 2 (*GJC2*) has emerged as a critical regulator of lymphatic function. *GJC2* encodes connexin 47 (Cx47), a transmembrane protein that forms gap junctions to facilitate the intercellular transfer of ions and small signaling molecules (<1 kDa), thereby enabling metabolic and electrical coupling. C×47 is highly expressed in lymphatic endothelial cells (LECs) and is essential for synchronizing depolarization to drive the coordinated lymphangion pumping required for fluid propulsion. *GJC2* mutations disrupt this electrochemical coupling, leading to uncoordinated pumping and functional obstruction, and typically segregate in an autosomal dominant manner with variable age of onset. However, the specific mechanisms by which different *GJC2* domains affect channel gating or assembly, and how these correlate with phenotypic severity, remain areas of active investigation.

To expand the genetic spectrum of hereditary PL, whole exome sequencing (WES) was employed to analyze a multigenerational Chinese family pedigree where four-limb PL segregates in an autosomal dominant manner. Our analysis identified a novel pathogenic variant in *GJC2*. Importantly, we also detected a novel co-segregating variant in *OBSCN* (Obscurin), a gene located in close physical proximity to *GJC2* on chromosome 1q42.13. *OBSCN* encodes a giant cytoskeletal signaling protein previously linked to cardiomyopathies and skeletal muscle channelopathies [4]. Although *OBSCN* has not been previously associated with the lymphatic system, its structural roles in cytoskeletal organization and signaling functions via rho guanine nucleotide exchange factor (RhoGEF) domains make it a biologically plausible candidate for endothelial dysfunction.

We hypothesize that the high linkage disequilibrium (LD) between *GJC2* and *OBSCN* in this pedigree has preserved a pathogenic haplotype. We propose that the *OBSCN* variant may act as a genetic modifier, compromising the cytoskeletal integrity of LECs and exacerbating the functional deficit caused by the *GJC2* gap junction dysfunction. This study presents the first clinical and molecular evidence linking *OBSCN* to primary lymphedema, expanding the genetic spectrum and underscoring the complexity of genetic interactions in hereditary lymphatic disorders.

## Methods

### Family recruitment

This study involved a comprehensive genetic and clinical evaluation of a four-generation Chinese family consisting of 26 members. The study protocol was reviewed and approved by the Ethics Committee of Dongzhimen Hospital (Approval No. DZMEC-KY-2019-110). All procedures were conducted in strict accordance with the Declaration of Helsinki. Written informed consent was obtained from all participating subjects; for individuals under the age of 18, written consent was provided by their parents or legal guardians. Detailed medical histories were collected for all 26 members. The pedigree analysis indicated an autosomal dominant pattern of inheritance. A subset of the family, comprising members critical for linkage analysis, was selected for WES. This subset (*n* = 8) included five affected individuals (I-4, II-15, II-16, II-26, III-20) and three unaffected or clinically indeterminate individuals (II-8, II-13, III-17) to serve as intra-familial controls (Fig. 2).

### Clinical evaluation and lymphatic imaging

Phenotypic characterization was performed using a combination of physical examination and imaging. Lymphedema severity was graded according to the International Society of Lymphology (ISL) staging system: Stage I, early accumulation of fluid high in protein content that subsides with limb elevation (pitting may occur); Stage II, limb swelling that does not resolve with elevation alone, with the onset of connective tissue proliferation (fibrosis); Stage III, lymphostatic elephantiasis characterized by trophic skin changes, such as acanthosis and fat deposits.

To objectively assess lymphatic transport, the proband (II-26) underwent lymphangioscintigraphy of both the upper and lower extremities [5]. Technetium-99 m labeled dextran (99mTc-DX) was used as the tracer. A dose of 5 mCi per extremity was injected subcutaneously into the interdigital spaces between the first-second and fourth-fifth digits (fingers for upper limbs and toes for lower limbs). Whole-body planar imaging was performed at 10 minutes, 1 hour, 3 hours, and 6 hours post-injection to visualize the tracer uptake and transport. Additionally, venous duplex ultrasound was performed to exclude deep vein thrombosis or chronic venous insufficiency as primary causes of the edema.

### Whole Exome Sequencing (WES) pipeline

#### DNA extraction and library preparation

Genomic DNA was extracted using the QIAamp DNA Blood kit (Qiagen, 51104) according to the manufacturer’s instructions. DNA quantity and purity were assessed using a NanoDrop 2000 spectrophotometer (Thermo Fisher Scientific); samples were required to meet an OD260/OD280 ratio of 1.8–2.0 and a total mass > 2.0 μg. For library construction, 500–1000 ng of gDNA was mechanically sheared via sonication to generate fragments with a mean size of 250–300 bp. Fragment size distribution was verified by 2% agarose gel electrophoresis. The sheared DNA underwent end-repair, A-tailing, and adapter ligation using the NEBNext UltraExpress® DNA Library Prep Kit (NEB, #E3325S).

#### Hybridization capture and sequencing

Target enrichment was performed via liquid hybridization using the Agilent SureSelect Human All Exon V6 capture kit. This panel covers the coding regions (exons) of approximately 19,396 human genes, with a total target region size of 35.1 Mb. The enriched libraries were quantified using a Qubit 2.0 Fluorometer to ensure a concentration > 5 ng/μL. High-throughput sequencing was conducted on an Illumina NovaSeq 6000 platform (S4 flow cell) in Paired-End 150 bp (PE150) mode. The sequencing metrics aimed for an average depth of >100X, with >98% of the target bases covered at ≥20X to ensure high-confidence variant calling.

#### Bioinformatics and variant calling

The raw sequencing data (BCL files) were demultiplexed and converted to FASTQ format. Quality control was performed using Fastp to trim adapter sequences and filter low-quality reads (Phred score < Q30). The clean reads were aligned to the human reference genome (hg19/GRCh37) using the Burrows-Wheeler Aligner (BWA-MEM algorithm). Post-alignment processing, including sorting, indexing, and removal of PCR duplicates, was performed using SAMtools. Variant calling (SNPs and Indels) was executed using Sentieon Genomics Tools (v202010.04), a highly accelerated implementation of the GATK Best Practices pipeline. Variants with a sequencing depth < 6X were discarded to minimize false positives.

### Variant filtering and prioritization strategy

To identify the causal mutation, we applied a rigorous stepwise filtering strategy, specifically designed to address the heterogeneity of the disease and population structure:

#### Population frequency filtering

We excluded common polymorphisms by filtering against public population databases, including the 1000 Genomes Project (1000G), the Exome Aggregation Consortium (ExAC), the NHLBI Exome Sequencing Project (ESP6500), and gnomAD. Variants with a Minor Allele Frequency (MAF) > 0.001 (0.1%) in any of these databases were removed.

#### Segregation analysis

Based on the pedigree, we filtered for variants fitting an autosomal dominant inheritance model. We retained only those variants that were heterozygous in all five affected individuals and absent (wild-type) in the three unaffected intra-familial controls.

#### Pathogenicity and stability prediction

The functional impact of missense variants was assessed using multiple in silico algorithms: SIFT (Sorting Intolerant from Tolerant), PROVEAN, PolyPhen-2 (HVAR and HDIV models), and MutationTaster. Variants predicted to be “Tolerated” or “Benign” by the majority of these tools were deprioritized. Splice-site variants were evaluated using MaxEntScan to predict disruptions of 5‘or 3’ splice sites. To further assess the impact on protein thermodynamic stability, the change in folding free energy (ΔΔG) was predicted using the SAAFEC-SEQ web server, a sequence-based method employing gradient boosting decision trees and evolutionary features.

#### Gene function and disease association

We cross-referenced candidate genes with the OMIM (Online Mendelian Inheritance in Man), ClinVar, and HGMD (Human Gene Mutation Database) databases to prioritize genes with known roles in vascular development, cytoskeletal organization, or previously reported associations with lymphedema or cardiomyopathy.

### Validation via Sanger sequencing

Candidate variants identified by WES were validated using Sanger sequencing in all available family members (*n* = 26). This step was crucial to confirm co-segregation in the extended pedigree, including those not sequenced by WES. Specific primers flanking the mutation sites in *GJC2*, *OBSCN*, and *DNAH14* were designed using NCBI Primer-BLAST (Table 2). PCR amplification was performed using CloneAmp HiFi Polymerase (Takara). The amplicons were sequenced using the BigDye Terminator v3.1 Cycle Sequencing Kit on an ABI 3130xl Genetic Analyzer (Applied Biosystems). Sequencing data were analyzed using Chromas and aligned to the reference genomes.

### Linkage Disequilibrium (LD) analysis

To investigate the relationship between the *GJC2* and *OBSCN* variants, we analyzed the local haplotype structure. Single nucleotide polymorphism (SNP) data for the chromosomal region 1q42.13 were retrieved from the NCBI SNP database. Linkage disequilibrium (LD) blocks were constructed and visualized using haploview v0.4.2 (Broad Institute) with default settings. A window size of 500 kb was utilized to capture the extent of the haplotype block shared by the affected family members.

### Protein structure modeling and visualization

The three-dimensional structures of human C×47 and obscurin were analyzed using the PyMOL Molecular Graphics System (Version 2.5, Schrödinger, LLC). The wild-type structural models were obtained from the AlphaFold Protein Structure Database (AF-Q5T442-F1 for Cx47). To evaluate the structural impact of the novel variants, in silico mutagenesis was performed. For Cx47, glycine at position 96 was substituted with valine (p.Gly96Val) within the second transmembrane helix (TM2). We specifically analyzed the steric alignment of residue 96 with its spatial neighbors on the helical face, valine 92 (Val92) and histidine 100 (His100), to assess potential disruptions in helix packing and stability. For obscurin, the modeling encompassed amino acids 6341–7968. We examined the interaction network of the wild-type phenylalanine 7465 (Phe7465) and mutant isoleucine 7465 (Ile7465) with adjacent residues proline 7463 (Pro7463), glutamate 7468 (Glu7468), and valine 7486 (Val7486) to assess changes in local geometry and structural stability within the Ig-like domain.

## Results

### Clinical characteristics and variable expressivity

**Fig. 1:**
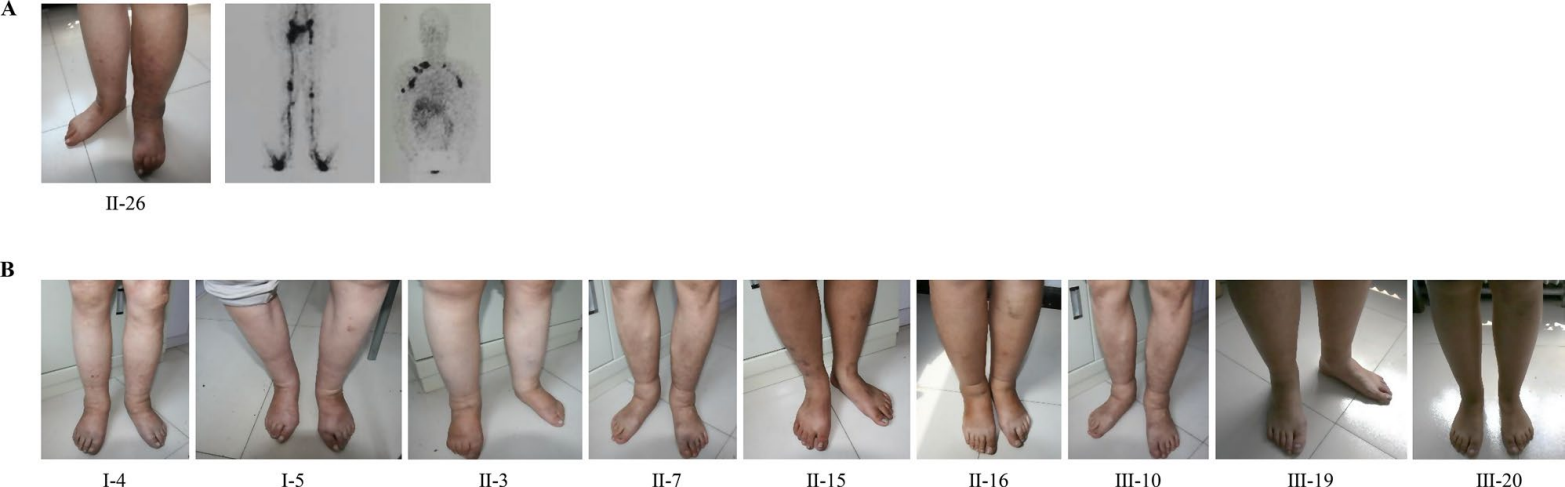
Clinical presentation and lymphatic imaging of affected family members. (**A**) Clinical phenotype and imaging findings in the proband (II-26). Left panel: photograph showing Stage III lymphedema (elephantiasis) with marked skin changes in the lower extremities. Right panels: lymphoscintigraphy demonstrating systemic lymphatic dysfunction, characterized by bilateral lymphatic hypoplasia in both upper and lower limbs, non-visualization of the left venous angle, and extensive dermal backflow. (**B**) Representative photographs illustrating primary bilateral lower limb lymphedema in individuals I-4, I-5, II-3, II-7, II-15, II-16, III-10, III-19, and III-20

The proband (II-26), a 60-year-old male, presented with chronic edema involving both lower and upper extremities, with symptoms beginning at age 16. Physical examination revealed stage III lymphedema with marked skin thickening (pachydermia) and onychauxis (thickened toenails). Lymphoscintigraphy demonstrated severe lymphatic dysfunction, including bilateral lymphatic hypoplasia in the lower and upper limbs, non-visualization of the left venous angle (suggesting obstruction of the thoracic duct or its subclavian connection), and extensive dermal backflow, a hallmark of retrograde lymphatic flow (Fig. 1A).

**Fig. 2:**
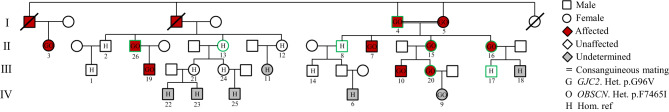
Pedigree of the family demonstrating autosomal dominant lymphedema and segregation of candidate variants. Squares represent males; circles represent females. Clinical status: red filled symbols indicate affected individuals; open symbols indicate unaffected members; grey filled symbols denote individuals with undetermined clinical status (age < 15 years). A double horizontal line indicates consanguinity (between I-4 and I-5). Genotyping: individuals selected for whole exome sequencing (WES) are highlighted with green outlines. Genotypes are annotated as follows: G, heterozygous for *GJC2* c.287G > T; O, heterozygous for *OBSCN* c.22393T > A; H, homozygous for the reference allele (wild-type)

Family history revealed a high penetrance of lymphedema across four generations (Fig. 2). Notably, a consanguineous marriage (between I-4 and I-5) was documented in the first generation. Due to the invasive nature of the procedure, lymphoscintigraphy was restricted to the proband (II-26); diagnoses of other family members were established based on clinical history and physical examination (Fig. 1B). Of the 26 members assessed, 10 were clinically diagnosed with PL. Among the remaining 16 unaffected members, 9 were over 15 years of age and clinically healthy; the other 7 were under 15 years old, and given the variable age of onset, their clinical status remains undetermined (Supplementary Table 1).

The phenotype was predominantly characterized by lower limb swelling, particularly below the knee, but showed marked variability in age of onset and severity (Table 1): **(I) Variable Onset**: while the majority of affected individuals (e.g., I-4, I-5, II-15, II-16, III-20) developed symptoms during adolescence (15–20 years), consistent with lymphedema praecox, significant deviations were observed. Individual II-7 presented with congenital lymphedema (onset at birth), whereas individual II-3 developed symptoms in childhood (age 8). **(II) Disease Severity and Complications:** severity ranged from stage I (mild edema) in younger members to stage III (elephantiasis) in the proband. Complications were not restricted to a specific age or gender but were observed in several individuals. Recurrent lymphangitis was documented in individuals I-4, II-3, and II-26. Varicose veins were present in II-7, II-26, and III-10. Additionally, onychauxis (thickened toenails) was observed in II-3 and II-26. **(III) Regional Involvement:** while lower limb edema was universal, upper limb involvement was present in three cases with longer disease duration (II-26, II-3, and I-4), supporting a systemic lymphatic defect consistent with a four-limb lymphedema syndrome.

**Table 1:** Clinical and genetic characteristics of the pedigree

Pedigree	PL	Age (current)	Age of onset	ISL stage	Lymphoscintigraphy findings	Presentation	Other	Sanger Sequencing	
								*GJC2* c.287G > T	*OBSCN* c.22393T > A
I-4	A	80	15–20	II	Not performed	4-Limb edema	Lymphangitis	Het	Het
I-5	A	81	15–20	I	Not performed	Bilateral lower limb		Het	Het
II-3	A	65	8	II	Not performed	4-Limb edema	Lymphangitis; onychauxis (thickened toenails)	Het	Het
II-7	A	54	Congenital	I	Not performed	Bilateral lower limb	Varicose veins	Het	Het
II-15	A	49	15–20	I	Not performed	Bilateral lower limb		Het	Het
II-16	A	47	15–20	I	Not performed	Bilateral lower limb		Het	Het
II-26	A	60	15–20	III	Bilateral lymphatic hypoplasia; non-visualization of left venous angle; dermal backflow	4-Limb edema	Lymphangitis; onychauxis (thickened toenails); varicose veins	Het	Het
III-10	A	20	15–20	I	Not performed	Bilateral lower limb	Varicose veins	Het	Het
III-19	A	28	15–20	I	Not performed	Bilateral lower limb		Het	Het
III-20	A	25	15–20	I	Not performed	Bilateral lower limb		Het	Het
IV-9	UnD	1						Het	Het

PL = primary lymphedema; ISL = International Society of Lymphology; PL status: A = affected; U = unaffected; UnD = clinical status undetermined due to young age; Sanger Sequencing: Het = heterozygous variant

Note: 1. International Society of Lymphology (ISL) staging system: Stage I (early accumulation of fluid that subsides with limb elevation); Stage II (limb swelling that rarely reduces with elevation alone, pitting is manifest); Stage III (lymphostatic elephantiasis with skin changes such as acanthosis, fat deposits, and warty overgrowths). 2. Imaging: Lymphoscintigraphy was performed only on the proband (II-26) for diagnostic confirmation; other affected members were diagnosed based on clinical history and physical examination

### WES identification of co-segregating variants

WES was performed on genomic DNA from eight selected family members, including five affected individuals and three unaffected controls (Fig. 2). The sequencing yielded high-quality data with >99% coverage of the exome. Based on the pedigree analysis indicating an autosomal dominant mode of inheritance, we applied a stepwise filtering strategy to identify candidate variants. We prioritized variants that exhibited strict co-segregation with the phenotype-specifically, those present in all five affected individuals and absent in the three unaffected subjects. This list was further filtered to exclude common polymorphisms reported in the 1000 Genomes Project database (MAF < 0.001). This rigorous process narrowed the candidates to 43 variants, which were then manually curated based on functional relevance to lymphatic or vascular development.

Three novel heterozygous missense variants were identified as the most promising candidates. First, a c.287G > T (p.Gly96Val) variant was detected in exon 2 of *GJC2* (NM_020435), a well-established cause of autosomal dominant hereditary lymphedema [6]. Second, a c.22393T > A (p.Phe7465Ile) variant was identified in exon 56 of *OBSCN* (NM_001098623); this gene encodes obscurin, a giant sarcomeric protein recently implicated in cardiomyopathy, suggesting a potential structural role in lymphatic muscle cells [7]. Third, a c.2060A > T (p.Asp687Val) variant was found in *DNAH14*, a gene that regulates ciliary movement, with mutations associated with ciliopathies that may overlap with lymphatic defects [8]. These three candidate variants were selected for further validation in all available family members using sanger sequencing.

### Validation and in silico analysis

Sanger sequencing was performed on all 26 available family members to validate the segregation of the three candidate variants, using specific primers listed in Table 2. This analysis led to the exclusion of *DNAH14*, as the variant was absent in affected individual III-19, indicating it was not the causal gene. In contrast, the variants in *GJC2* (c.287G > T) and *OBSCN* (c.22393T > A) demonstrated co-segregation with the disease phenotype. All 10 clinically affected individuals were heterozygous for both variants, whereas the 15 clinically unaffected members were homozygous for the wild-type alleles (Supplementary Table 1). Notably, individual IV-9, a currently asymptomatic 1-year-old female, was found to carry both heterozygous variants. Given the typical adolescent onset observed in this pedigree, she is considered as a pre-symptomatic carrier with a high genetic risk for developing lymphedema in the future.

**Table 2:** Primer sequences and conditions used to amplify

Name of primer	Sequence (5’–3’)	Annealing temp (°C)	Product size (bp)
GJC2-F	GGAGGAGATCCACAACCA	58.5	673
GJC2-R	GAAGCCGTACAGCAGGTA		
OBSCN-F	GAGGAGGTGATGGTGGTA	57	459
OBSCN-R	TGGCAGAGATGAGGACAC		
DNAH14-F	TAGCCACAATTACTCCTCTT	57	422
DNAH14-R	AACAACTGACGAGCCTAC		

**Fig. 3:**
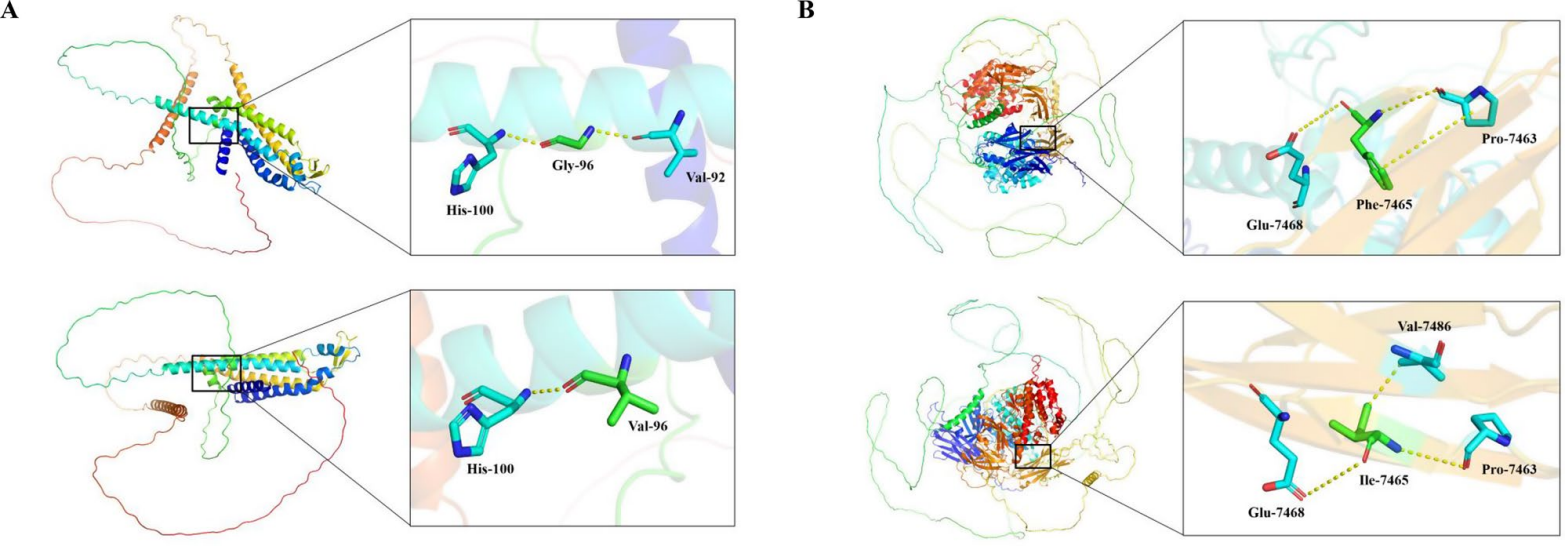
Structural modeling of the C×47 and obscurin candidate variants. (**A**) The C×47 p.Gly96Val variant. Ribbon representation of the C×47 protein with a zoomed-in view of the second transmembrane domain (TM2). Top panel (wild-type): the native glycine residue (Gly96) possesses a minimal side chain, allowing for tight packing against both valine 92 (Val92) and histidine 100 (His100) (indicated by yellow dashed lines). This dual contact stabilizes the helical packing. Bottom panel (mutant): the p.Gly96Val mutation introduces a bulky valine residue (Val96). Due to steric constraints, Val96 retains the contact with His100 but loses the interaction with Val92. This loss of molecular contact is predicted to compromise the thermodynamic stability of the transmembrane domain. (**B**) The obscurin p.Phe7465Ile variant. The model encompasses amino acids 6341–7968 of the obscurin protein. Top panel (wild-type): the native phenylalanine residue (Phe7465) contains an aromatic side chain that forms stabilizing molecular interactions with proline 7463 (Pro7463) and glutamate 7468 (Glu7468) (yellow dashed lines). Bottom panel (mutant): the mutation substitutes phenylalanine with isoleucine (Ile7465). The aliphatic side chain of isoleucine alters the local geometry; while it maintains contacts with Pro7463 and Glu7468, it also forms a novel, likely aberrant interaction with valine 7486 (Val7486). This alteration in the interaction network suggests a disruption of the native structural Fold in this region

Bioinformatics tools and detailed structural modeling were utilized to assess the pathogenicity of these confirmed variants. Structural modeling (Fig. 3A) revealed that the wild-type glycine residue in C×47 possesses a minimal side chain, facilitating stable molecular interactions with both Val92 and His100 that stabilize the helical packing of the second transmembrane domain (TM2). The p.Gly96Val mutation introduces a bulky valine residue; while Val96 retains contact with His100, steric constraints lead to the loss of the critical interaction with Val92. This loss of molecular contact is predicted to compromise the thermodynamic stability of the transmembrane domain (ΔΔG = −1.77 kcal/mol), likely interfering with membrane integration or connexon pore assembly (Table 3). For obscurin (Fig. 3B), the wild-type phenylalanine residue within the Ig-like domain 55 forms stabilizing interactions with Pro7463 and Glu7468. The p.Phe7465Ile mutation substitutes the aromatic side chain with an aliphatic one, altering the local geometry. While Ile7465 maintains contacts with Pro7463 and Glu7468, it forms a novel, likely aberrant interaction with Val7486. This alteration in the interaction network suggests a disruption of the native structural fold in this region (Table 3), which is supported by thermodynamic instability predictions (ΔΔG = −1.67 kcal/mol).

**Table 3:** Prediction of protein functional and stability outcomes of point mutation

	SIFT score	SIFT pred	Polyphen2 HDIV score	Polyphen2 HDIV pred	Mutation Taster score	Mutation Taster pred	ΔΔG
Cx47 (p.G96V)	0	D	1	D	1	D	−1.77
Obscurin (p.F7465I)	0.25	T	0.998	D	1	D	−1.67

D = deleterious; T = tolerated; pred = prediction

### Linkage disequilibrium analysis

**Fig. 4:**
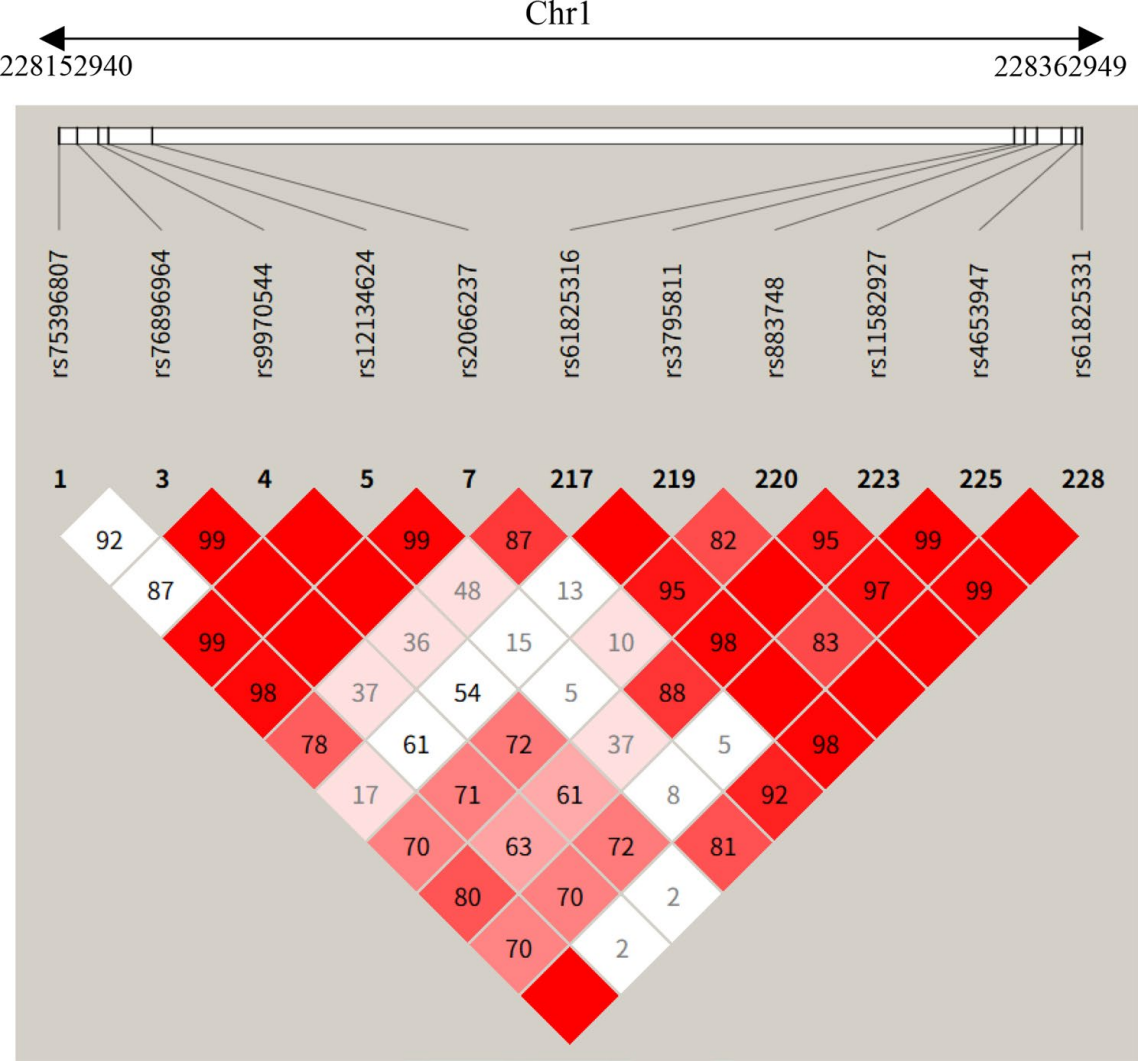
Linkage disequilibrium (LD) plot of the genomic region spanning *GJC2* and *OBSCN* on chromosome 1. The analysis covers a 210 kb interval (genomic coordinates: 228,152,940–228,362,949) containing SNPs associated with *GJC2* (left) and *OBSCN* (right). Pairwise LD values are visualized in the diamond matrix. Red diamonds indicate strong linkage disequilibrium (high r^2^ values), while the numbers within the squares represent r^2^ values expressed as percentages (e.g., 99 corresponds to r^2^ = 0.99). The extensive red block pattern demonstrates that the variants in *GJC2* and *OBSCN* reside within a shared haplotype block, supporting their co-inheritance in this pedigree

The *GJC2* and *OBSCN* genes are located on chromosome 1q42.13, separated by a physical distance of approximately 210 kb (Table 4). Haplotype analysis of SNPs in this region confirmed that these two variants reside within a shared haplotype block in this family (Fig. 4). The strong linkage disequilibrium (LD) suggests that these variants have been co-inherited through generations as a single genetic unit, rather than arising from independent mutational events.

**Table 4:** SNP positions used in linkage disequilibrium (LD)

SNP	Location	Gene	Distance (bp)
rs75396807	228152940	*GJC2*	5100
rs76896964	228156677	*GJC2*	1363
rs9970544	228160979	*GJC2*	−2939
rs61825316	228349074	*OBSCN*	14206
rs4653947	228361708	*OBSCN*	1572
rs61825331	228362949	*OBSCN*	331
rs3795811	228351267	*OBSCN*	12013
rs883748	228353736	*OBSCN*	9544
rs11582927	228358786	*OBSCN*	4494
rs12134624	228163073	*OBSCN*	207

“Distance” shows the length between the SNP and the mutation site of the gene where it is located

## Discussion

In this study, we performed a comprehensive genetic and clinical analysis of a multigenerational Chinese family affected by autosomal dominant PL. Following WES analysis of a subset of individuals, a novel mutation in *GJC2* (c.287G > T, p.Gly96Val) and a mutation in *OBSCN* (c.22393T > A, p.Phe7465Ile)—a gene not previously implicated in lymphedema—were identified. Both variants co-segregated with the disease phenotype and were predicted to have destabilizing effects on protein structure. This marks the first documented association between *OBSCN* and PL, thus expanding our understanding of the genetic underpinnings of this complex disease.

Genetic studies examining PL have identified approximately 23 associated genes or loci, validated through co-segregation in pedigrees and/or functional in vitro and/or in vivo analyses [9–11]. Furthermore, variants in an additional 22 genes have been implicated as causal, but lack definitive pathogenicity evidence [9, 12]. In the present study, WES effectively excluded pathogenic mutations in these known PL-associated genes. Consequently, the identification of the co-segregating *GJC2* and *OBSCN* variants suggests a novel molecular etiology. These findings indicate that these novel variants likely contribute to PL pathogenesis, particularly within the Chinese population, and highlight the value of WES in resolving genetically undiagnosed cases.

The *GJC2* gene, encoding the connexin 47 (Cx47) protein, is a recognized contributor to lymphatic dysfunction. Connexins are integral membrane proteins characterized by four transmembrane domains (TM1–TM4), two extracellular loops, and cytoplasmic *N*- and C-termini. The TMs are structurally critical for the oligomerization of six connexin monomers into a hemichannel (connexon) and for the correct insertion of this complex into the lipid bilayer [13–15]. In the study, the p.Gly96Val mutation is located in the TM2 domain. Structural modeling indicates the mechanism of destabilization: the mutation eliminates the critical stabilizing interaction between residue 96 and Val92 within the TM2 helix. This loss of contact, combined with the introduction of a bulky hydrophobic valine side chain, sterically hinders proper helix packing, likely leading to protein misfolding or retention in the endoplasmic reticulum (ER). Functionally, C×47 is critical for gap junctional communication between lymphatic endothelial cells, including the passage of ions and second messengers. The loss of functional surface C×47 diminishes intercellular coupling, disrupting the electrical network of the lymphatic vessel wall and preventing the coordinated propagation of calcium waves required for effective lymphangion contraction.

A hallmark challenge in interpreting *GJC2*-related lymphedema is the significant variability in penetrance and phenotypic severity. In this family, clinical onset ranged widely from congenital forms to childhood and adolescence, despite an identical *GJC2* background. This marked phenotypic heterogeneity strongly suggests that other genetic modifiers are influencing the clinical expression of the disease. Here, we propose that the co-segregating *OBSCN* variant (p.Phe7465Ile) functions as such a genetic modifier. *OBSCN* is canonically recognized for its structural role in different forms of skeletal and cardiac myopathies [16–21], but its expression and function are not restricted to muscle tissues. Obscurin may exert a relevant, albeit underappreciated, influence on endothelial biology through its functional domains—particularly the RhoGEF and Ig-like regions—which are integral to cytoskeletal organization [22].

Based on these findings, we further proposed a mechanistic model. The O*BSCN* variant likely exacerbates lymphatic dysfunction through dual structural and signaling pathways. Similar to muscle cells, lymphatic endothelial cells (LECs) require a robust cytoskeleton to withstand oscillating hydrostatic pressures. Obscurin, via its multiple Ig-like and FnIII domains, acts as a molecular spring and scaffold [23]. The structural modeling suggests that the p.Phe7465Ile mutation destabilizes this architecture by altering local geometry and forming an aberrant interaction with Val7486, which may perturb the native fold of Ig-like domain 55. This structural disruption may render LECs more fragile under mechanical stress. Beyond this scaffolding function, obscurin likely regulates valve morphogenesis via its RhoGEF domains, which activate RhoA—a master regulator of the endothelial actin cytoskeleton. Since RhoA signaling is essential for LEC alignment in response to shear stress and for the maintenance of lymphatic valves, any impairment in RhoGEF activity or localization caused by the variant could therefore lead to defective valve formation [24].

Based on these observations, we propose a synergistic pathogenicity model for this pedigree. In this scenario, the *GJC2* mutation creates a primary functional defect (lack of intercellular coordination), while the co-segregating *OBSCN* mutation imposes a secondary structural vulnerability (cytoskeletal fragility). The convergence of these two defects likely drives the high-penetrance, severe phenotype involving all four limbs observed in this family. The variability in onset age (from birth to adolescence) may then be explained by the cumulative effect of environmental triggers, such as minor trauma or infection, acting upon this genetically compromised background.

The close physical proximity of *GJC2* and *OBSCN* (~205 kb) on 1q42.13 offers a plausible structural explanation for our results. The observed co-segregation of these variants is consistent with physical linkage within a region of strong linkage disequilibrium (LD). This pathogenic haplotype may have originated in a common ancestor (founder effect). From an evolutionary perspective, the retention of this haplotype implies that neither variant alone was lethal or subject to strong purifying selection; rather, their potential synergistic combination may drive the severe disease phenotype. These findings highlight the importance of considering multi-genic contributions in WES data, especially for variants in close proximity that might functionally interact.

## Conclusions

In summary, this study implicates a novel *GJC2/OBSCN* haplotype as a putative driver of primary lymphedema in a Chinese pedigree. We provide a mechanistic rationale for the involvement of obscurin based on its cytoskeletal and signaling functions. These findings suggest that *OBSCN* should be considered a candidate gene in lymphedema panels, particularly for cases that are negative for standard markers or exhibit atypical severity. Future functional studies using LEC models or zebrafish knockouts are warranted to experimentally validate the synergistic crosstalk between C×47 and obscurin in lymphatic development. Elucidating these complex genetic interactions is pivotal for accurate genetic counseling and the development of precision therapeutic strategies for patients with refractory lymphedema.

## Electronic supplementary material

Below is the link to the electronic supplementary material.

Supplementary Material 1

## Data Availability

All relevant data and materials supporting the findings of this study are available from the corresponding author upon reasonable request.
