# Supplementary material for: Novel GJC2 and OBSCN variants co-segregating in a Chinese primary lymphedema pedigree
Source: Orphanet J Rare Dis. 2026 Jan 13;21:17. doi: 10.1186/s13023-026-04196-7 (PMC12809928; doi:10.1186/s13023-026-04196-7)
Supplement: Supplementary file 1 — Supplementary Material 1 [file 13023_2026_4196_MOESM1_ESM.docx]

Supplementary Table 1. Clinical findings and Sanger sequencing results for examined affected and unaffected family members.

| Pedigree | WES | PL | Gender | Age | Other | Sanger Sequencing | | |
| --- | --- | --- | --- | --- | --- | --- | --- | --- |
|  |  |  |  |  |  | *GJC2* c.287G>T | *OBSCN* c.22393T>A | *DNAH14*  c.2060A>T |
| III-1 | N | U | M | 35 |  | WT | WT | WT |
| II-2 | N | U | M | 63 |  | WT | WT | WT |
| II-3 | N | A | F | 65 | L, O | Het | Het | Het |
| I-4 | Y | A | M | 80 | L | Het | Het | Het |
| I-5 | N | A | F | 81 |  | Het | Het | Het |
| IV-6 | N | UnD | M | 2 |  | WT | WT | WT |
| II-7 | N | A | M | 54 | VV | Het | Het | Het |
| II-8 | Y | U | M | 57 |  | WT | WT | WT |
| IV-9 | N | UnD | F | 1 |  | Het | Het | Het |
| III-10 | N | A | M | 20 | VV | Het | Het | Het |
| III-11 | N | UnD | F | 7 |  | WT | WT | WT |
| II-12 | N | U | F | 54 |  | WT | WT | WT |
| II-13 | Y | U | F | 59 | VV | WT | WT | WT |
| III-14 | N | U | M | 33 |  | WT | WT | WT |
| II-15 | Y | A | F | 49 |  | Het | Het | Het |
| II-16 | Y | A | F | 47 |  | Het | Het | Het |
| III-17 | Y | U | M | 23 |  | WT | WT | WT |
| III-18 | N | UnD | M | 14 |  | WT | WT | WT |
| III-19 | N | A | M | 28 |  | Het | Het | WT |
| III-20 | Y | A | F | 25 |  | Het | Het | Het |
| III-21 | N | U | F | 37 |  | WT | WT | WT |
| IV-22 | N | UnD | M | 14 |  | WT | WT | WT |
| IV-23 | N | UnD | M | 8 |  | WT | WT | WT |
| III-24 | N | U | F | 27 |  | WT | WT | WT |
| IV-25 | N | UnD | M | 7 |  | WT | WT | WT |
| II-26 | Y | A | M | 60 | L, O, VV | Het | Het | Het |

WES = whole exome sequencing; PL = primary lymphedema; M = male; F = female; WES status: Y = WES performed; N = WES not performed; PL status: A = affected; U = unaffected; UnD = clinical status undetermined due to young age; Other clinical signs: L = lymphangitis; VV = varicose veins; O = onychauxis (thickened toenails); Sanger Sequencing: Het = heterozygous variant; WT = wild type (no mutation detected)
